# Post-9/11 Peripheral Neuropathy Symptoms among World Trade Center-Exposed Firefighters and Emergency Medical Service Workers

**DOI:** 10.3390/ijerph16101727

**Published:** 2019-05-16

**Authors:** Hilary L. Colbeth, Rachel Zeig-Owens, Mayris P. Webber, David G. Goldfarb, Theresa M. Schwartz, Charles B. Hall, David J. Prezant

**Affiliations:** 1 Fire Department of the City of New York, Bureau of Health Services, 9 Metrotech Center, Brooklyn, NY 11201, USA; Hilary.Colbeth@fdny.nyc.gov (H.L.C.); Mayris.Webber@fdny.nyc.gov (M.P.W.); David.Goldfarb@fdny.nyc.gov (D.G.G.); Theresa.Schwartz@fdny.nyc.gov (T.M.S.); David.Prezant@fdny.nyc.gov (D.J.P.); 2 Department of Medicine, Pulmonology Division, Montefiore Medical Center, Bronx, NY 10467, USA; 3 Department of Epidemiology and Population Health, Albert Einstein College of Medicine, Bronx, NY 10461, USA; charles.hall@einstein.yu.edu; 4 Department of Epidemiology and Population Health, Montefiore Medical Center, Department of Epidemiology and Population Health, Bronx, New York, NY 10467, USA; 5 Saul R. Korey Department of Neurology, Albert Einstein College of Medicine, Bronx, NY 10461, USA; 6 Department of Medicine, Pulmonology Division, Albert Einstein College of Medicine, Bronx, NY 10461, USA

**Keywords:** peripheral neuropathy, prevalence, World Trade Center, rescue/recovery workers, occupational exposure

## Abstract

Peripheral neuropathy can result from numerous conditions including metabolic disorders, inflammatory disease, or exposure to environmental or biological toxins. We analyzed questionnaire data from 9239 Fire Department of the City of New York (FDNY) World Trade Center (WTC)-exposed firefighters and emergency medical service workers (EMS) to evaluate the association between work at the WTC site and subsequent peripheral neuropathy symptoms using the validated Diabetic Neuropathy Symptom (DNS) score. We grouped the population into an “Indicated” group with conditions known to be associated with paresthesia (*N* = 2059) and a “Non-Indicated” group without conditions known to be associated (*N* = 7180). The level of WTC exposure was categorized by time of arrival to the WTC. Overall, 25% of workers aged 40 and older reported peripheral neuropathy symptoms: 30.6% in the Indicated and 23.8% in the Non-Indicated groups, respectively. Multivariable logistic models performed on the Non-Indicated group, and on the Non-Indicated in comparison with non-WTC exposed National Health and Nutrition Examination Survey (NHANES), found that the highest level of WTC-exposure was significantly associated with DNS positive outcomes, after controlling for potential confounders. In conclusion, this study suggests that symptoms of peripheral neuropathy and paresthesias are common and are associated with WTC-exposure intensity.

## 1. Introduction

The collapse of the World Trade Center (WTC) buildings in New York City after the terrorist attacks on 11 September, 2001 (9/11) resulted in high volumes of aerosolized dust and gases including neurotoxins such as lead, aluminum, cadmium, manganese, tin, and complex hydrocarbons, specifically polychlorinated biphenyl (PCB), dioxins, and polycyclic aromatic hydrocarbons (PAHs) [1,2]. Additionally, the 10-month rescue/recovery effort may have exposed workers to organic solvents [3] and pesticides [4], which are known causes of peripheral neuropathy and neurodegenerative diseases [1].

The peripheral nervous system may be particularly vulnerable to environmental and metabolic insult that can result in peripheral neuropathy (PN), which can take axonal, demyelinating, or mixed forms [5]. Diabetic neuropathy is most common [6,7], but there are other types, such as inflammatory and toxic. Inflammatory neuropathies include those that are infectious, for example, those of autoimmune origin like sarcoidosis and Gullian–Barré syndrome [5,8]. Toxic neuropathies are often related to chemotherapeutic agents (chemotherapy-induced toxic peripheral neuropathy) [9,10], chronic alcohol abuse [11,12], or exposure to heavy metals and other environmental toxins [13], which result in primary axonal damage. Toxins may also include industrial solvents due to their use of hexacarbons [14], a group of chemical compounds shown to cause length-dependent distal sensory loss, weakness, atrophy, reduced distal reflexes, and autonomic dysfunction.

To date, three previous questionnaire-based studies have investigated the prevalence and risk factors for peripheral neuropathy in smaller WTC-exposed populations and reported that neuropathy symptoms were commonly associated with WTC-related exposure or WTC cleaning work [15,16,17]. The purpose of the current study is to investigate the prevalence of self-reported peripheral neuropathic symptoms and paresthesias among more than 9000 WTC-exposed Fire Department of the City of New York (FDNY) firefighters and emergency medical workers (EMS) between 7 June 2017, when the Diabetic Neuropathy Symptom (DNS) score was added to FDNY monitoring questionnaires, and 6 April 2019. The DNS score was developed by an expert panel consisting of a diabetologist, a vascular internist, a neurologist, and a physician for rehabilitation medicine seeking a validated, rapid, and easy to preform screening tool. The score demonstrates good inter-rater agreement (Cohen’s weighted κ for both raters was 0.89 and 0.78), reliability (0.64) and sound screening properties due, in particular, to its high sensitivity (79%) and specificity (78%) [18]. We hypothesized that WTC exposure, overall, and specifically, arrival time to the disaster site would be associated with a higher prevalence of neuropathic symptoms both as measured by the DNS and by paresthesias of the lower and upper extremities. Further, we included as a comparison population, data from non-WTC individuals who participated in the 2003–2004 National Health and Nutrition Examination Survey (NHANES) survey [19].

## 2. Materials and Methods

### 2.1. Data Collection

The FDNY WTC Health Program performs periodic health evaluations on all active FDNY members, and on WTC-exposed retired members, both firefighters and EMS, every 12–18 months. Since 2001, self-administered health questionnaires have been used to collect information about WTC exposure, health behaviors such as smoking history, and physician diagnoses.

### 2.2. Description of the Study Population

The source population consisted of 14,185 WTC-exposed firefighters and EMS enrolled in FDNY WTC Health Program (WTCHP) who provided written consent for research. The inclusion criteria for this study were: (1) having arrived at the disaster site between the morning of 11 September, 2001 (9/11) and 24 September, 2001; (2) being an active FDNY firefighter or EMS on 9/11; (3) having taken at least one post-9/11 health questionnaire within the study period; and, (4) being 40 years or older at the time of their most recent questionnaire. The final study population included 9239 firefighters and EMS; this population was separated into two groups. The first, hereafter called the ‘Indicated’ group, was comprised of those with conditions that are known to be linked to peripheral neuropathy (*N* = 2059), which include those with: diabetes (glucose ≥140 mg/dL on the most recent FDNY blood test), a history of confirmed cancer (not including in situ cancers or non-melanoma skin cancers) [20,21,22], or a history of a confirmed autoimmune disease (including phospholipid, aplastic, myositis, lupus, sclerosis, myasthenia, psoria, rheum, sarcoidosis, Sjögren’s, thrombocytopenia, and Wegener’s) [23,24]. The second, hereafter called the ‘Non-Indicated’ group, was comprised of those without such conditions (*N* = 7180). These groups are mutually exclusive.

The Montefiore Medical Center/Albert Einstein College of Medicine’s Institutional Review Board approved this study (IRB: 07-09-320).

### 2.3. Defining WTC Exposure Intensity

WTC exposure is defined from the earliest post-9/11 health questionnaire. We categorized exposure based on the FDNY-WTC exposure intensity index [25] as being high (arrived on the morning of 9/11), moderate (arrived afternoon of 9/11 or on 9/12/2001), or low (arrived between 9/13/2001 and 9/24/2001) [25,26].

### 2.4. Defining FDNY Peripheral Neuropathy Symptoms

Self-administered health questionnaires completed by all FDNY firefighters and EMS at their monitoring exams captured self-reported, physician-diagnosed peripheral neuropathy, and beginning in 2017, the validated four-item DNS score was used for diagnosing distal diabetic polyneuropathy [18]. Each DNS item scores one point, a score of 1 or greater signals the presence of polyneuropathy. Additionally, FDNY added the following two questions: “Considering any time in the last two weeks (excluding during exercise), do you have prickling, pins and needles, burning, aching pain or tenderness in your legs or feet?” followed by the same question regarding the arms or hands. Answer choices were “never, occasional, often, or almost continuously”. All analyses used information from each participant’s most recent questionnaire within the study period.

### 2.5. Defining NHANES Peripheral Neuropathy Symptoms

The 2003–2004 National Health and Nutrition Examination Survey (NHANES) [19] (*N* = 3299) asked participants 40 years and older about numbness, loss of feeling, painful sensation, or tingling in the hands or feet, other than from the hands or feet falling asleep, in the past three months. Survey participants who confirmed such experiences in their feet or both their hands and feet were identified as having symptoms of peripheral neuropathy. We excluded 519 who reported that a health professional had ever diagnosed them with “diabetes” or “sugar diabetes”, and 62 who reported either “borderline” or “don’t know” for diagnosed diabetes. The final population consisted of 2718 non-diabetic NHANES survey participants.

### 2.6. Statistical Methods

First, prevalences of self-reported peripheral neuropathy and symptoms were calculated among the Indicated and Non-Indicated groups, and a weighted prevalence was calculated for NHANES (via SAS PROC SURVEYMEANS) using the two-year interviewed sample weights provided in the NHANES data file.

Then we conducted two phases of analyses exclusively on the Non-Indicated group: (1) the association of WTC exposure and peripheral neuropathy and (2) the estimation of the likelihood of symptoms in the FDNY cohort relative to NHANES.

(1) Odds ratios (ORs) and 95% confidence intervals (CIs) were used to explore associations between peripheral neuropathic symptoms and various risk factors. Multivariable logistic regression was used to analyze the association between the level of WTC exposure and peripheral neuropathy as defined by the DNS. Further, we investigated the association with the frequency of paresthesia (often or almost continuously vs never or occasional) separately in the lower and upper extremities, and in the extremities compiled. Additionally, we tested linear trends in the exposure–response relationship of each model by including WTC exposure as an ordinal predictor. All multivariable models controlled for work assignment on 9/11 (EMS, firefighter), sex, race (non-Hispanic white, non-white (non-Hispanic black, Hispanic, other)), smoking history at the time of the questionnaire (ever, never), chronic alcohol abuse (yes, no), and age at exam. Chronic alcohol abuse, based on the Alcohol Use Disorders Identification Test (AUDIT) [27], was defined as positive for alcohol abuse on every questionnaire after the first ever indication.

(2) Using the analytic technique employed by Myers et al. [28], we created a combined analytic file for multivariable logistic regression estimation by normalizing variable names and variable coding for the NHANES and FDNY Non-Indicated datasets and appending the two data files. In the combined file, two-year interviewed sample weights formed the frequency weights for NHANES records, and FDNY records received a frequency weight of one. An indicator variable identified records that were NHANES or FDNY. In this way, the NHANES cohort is used as a national reference population to standardize the FDNY Non-Indicated group. Our models compared the association of peripheral neuropathic symptoms with the FDNY cohort overall and to each level of WTC exposure (via SAS PROC SURVEYLOGISTIC), adjusting for sex, race (non-Hispanic white, non-white (non-Hispanic black, Hispanic, other)), and age at exam. As a secondary analysis, a WTC exposure test for trend was conducted. In order to compare the DNS questions to NHANES, we removed the first DNS item which asks if a person is suffering from unsteadiness in walking; thus, a positive score on the DNS was restricted to a score of 1 or higher using only the other three items.

All analyses were conducted using SAS 9.4 (SAS Institute, Inc., Cary, NC, USA).

## 3. Results

### 3.1. Baseline Characteristics

The study cohort (*N* = 9239) was 97.6% male, 89.7% non-Hispanic white, and 88.2% were firefighters on 9/11. Most of the cohort (69.3%) was moderately-exposed to the WTC disaster. The mean (SD) age on 9/11 was 39.7 (7.5) years, and 25.3% (*N* = 2341) of the study cohort scored positive on the DNS. These demographic characteristics are reflected in the Indicated and Non-Indicated groups presented in Table 1. Compared with the Indicated, the Non-Indicated group was younger, on average, included a slightly higher percentage of non-Hispanic whites and firefighters, and had a greater proportion of never smokers.

### 3.2. Prevalence of Neuropathic Symptoms

The prevalence of scoring positive on the DNS among the Indicated group was 30.6% while the Non-Indicated group scored 23.8% (Table 2). Both groups, with 20.2% of the Indicated group and 15.2% of the Non-Indicated group, commonly reported symptoms of burning, aching pain or tenderness in the legs or feet. The greatest proportion of overlap between items in the Indicated group and Non-Indicated group, respectively, was by those who answered positively to questions 2–4 (19.8%; 16.1%); additionally, 72 (11.4%) of the Indicated group and 146 (8.5%) of the Non-Indicated group answered positively to all four questions. The Indicated group reported a greater prevalence of paresthesia in the legs or feet (11.7%, *N* = 240) than in the arms or hands (8.1%, *N* = 167), as did the Non-Indicated group (8.5%, *N* = 613; 7.1%, *N* = 509). Physician-diagnosed peripheral neuropathy was very rare: approximately 2% of the Indicated group and 1% of the Non-Indicated group.

### 3.3. Associations of 9/11-Related Exposures with Peripheral Neuropathy Symptoms

Final logistic regression models used the following outcomes: positive on the DNS; and often/almost continuously experiencing paresthesias in the legs or feet, in the arms or hands, or in both (Table 3). Those with the highest exposure to the WTC disaster (arriving the morning of 9/11) were more likely to score positive on the DNS (OR 1.35, 95% CI 1.10–1.65) than those in the lowest exposure group (*P* test for trend *P* = 0.004). This signal became stronger when assessing paresthesias, except for paresthesias of the legs or feet where the association was not significant. Trend tests between WTC exposure level and paresthesias of the arms and the extremities compiled, respectively, were significant (*P* test for trend *P* = 0.036; 0.006). There were no significant associations with work assignment on 9/11 (EMS vs Fire). Sex, older age, a history of smoking, and chronic alcohol abuse were associated with almost every outcome.

### 3.4. U.S. Population Prevalence and Comparison to FDNY Cohort

Among the 2003–2004 non-diabetic NHANES population, a weighted estimate of 13% (95% CI 11.2–15.1) reported experiencing symptoms of peripheral neuropathy, as compared with the estimated 23% (95% CI 19.0–27.1%) of the 519 diabetic survey participants. Our restricted DNS definition (DNS scored excluding the first question) reduced the prevalence of reported symptoms to 22.3% among the Non-Indicated group. The multivariable logistic regressions (Table 4) found that any exposure to the WTC increased the odds of reporting symptoms (OR 2.06, 95% CI 1.65–2.57). An exposure response gradient became apparent when each level of WTC exposure was compared to the NHANES population (*P* test for trend *P* < 0.0001) (Table 4).

## 4. Discussion

Our study uniquely examines peripheral neuropathy symptoms reported about 15 years after the inhalation and dermal absorption of WTC particulates and gases in a cohort of exposed firefighters and EMS. Nearly one-quarter of the Non-Indicated group reported one or more symptoms of peripheral neuropathy, compared with 31% of those with a comorbid condition known to be associated with paresthesias, specifically diabetes, cancer, or autoimmune disease. The prevalence of reported DNS symptoms was lower in the Non-Indicated than in the Indicated group, but not by half, a prevalence difference that has been demonstrated between non-diabetic and diabetic groups in the general population [19,29,30]. Not only does this result demonstrate the applicability of the DNS in our cohort, it also indicates a potential for WTC-exposed workers, other than those typically at risk, to be experiencing peripheral nerve damage. Our findings illustrated moderate associations between the most highly exposed workers (rescue/recovery workers most exposed to massive volumes of aerosolized particulates and pulverized building materials) and a positive DNS score and with paresthesias of the upper extremities. As expected, we found stronger associations with those who reported paresthesias of both the upper and lower extremities.

Unlike the previous two WTC-associated studies of paresthesia, we used peripheral neuropathic symptom data from the 2003–2004 NHANES non-exposed, non-diabetic population as a comparison. NHANES prevalence data from this period suggested that any WTC exposure, whether the most or the least exposed, increased by two-fold the likelihood of reporting neuropathic symptoms. This standardization to NHANES bolsters the connection between WTC exposure and paresthesia symptoms also found in previous neuropathy symptom studies [15,16,17]. Moreover, we used a validated screening questionnaire, the DNS, in addition to questions on paresthesias of the upper and lower extremities, similar to the questions used by Marmor et al. [16], to examine prevalence in our population using multiple measures; therefore, giving our findings a more robust framework. Further support for our findings can also be found in a 2016 study of 16 WTC-exposed responders and survivors, which concluded that there was a higher probability of a neuropathy diagnosis in patients who were WTC-exposed as compared with others referred for electromyography (EMG) testing [31].

Our study has some limitations. Since the FDNY WTCHP did not add questions about peripheral neuropathy symptoms to the monitoring questionnaires until 2017, we are not yet able to assess longitudinal persistence of paresthesias, which could help elucidate the long-term effects of the WTC on neurodegeneration in the peripheral nervous system. And, while we adjusted for work assignment on 9/11, sex, race, smoking history, chronic alcohol abuse, and age, our logistic models may not have fully controlled for unmeasured confounding. Additionally, during the second phase of analysis, we were unable to remove those who had a history of cancer or autoimmune disease from the NHANES dataset. We can assume, therefore, that the already strong association between WTC exposure and peripheral neuropathy symptoms was biased toward the null. We acknowledge that NHANES does not represent an exact counterfactual for our WTC-exposed cohort and, instead, a comparison group comprised exclusively of firefighters with no exposure to the disaster or a truly random sample of the U.S. population would be most desirable; nevertheless, no such cohort data were available at this time. Finally, the use of cross-sectional questionnaire data cannot establish causality, we cannot account for the specific impacts of career firefighting versus WTC-specific particulate exposures, and very few rescue/recovery workers reported a physician diagnosis of peripheral neuropathy. However, the high prevalence of symptoms in the Non-Indicated group and significant associations with WTC exposure intensity and as compared to the general population underscore the need for further investigation.

## 5. Conclusions

EMG, skin punch biopsy, and nerve conduction velocity testing will provide confirmation of neuropathies and determine the specific types of nerve injuries that may be associated with WTC exposure. The mechanism by which WTC exposure results in neuropathy will require further investigation by others. Such research has been started by Stecker et al.’s 2014 study on rat sciatic nerves [32], which provided biological plausibility for the effect of WTC exposure mediated by a methanol-soluble element in WTC dust, leading to an increased risk of neuropathy. Furthermore, recent studies of neurodegeneration, in the military and populations exposed to terrorist attacks other than the WTC, have begun to stress the relevance of toxic neuropathy [33,34,35]. In conclusion, our study suggests that WTC exposure may result in an increased risk of peripheral neuropathy in rescue/recovery workers, especially those who were most highly exposed to the dust cloud. This risk appears to be independent of the illnesses and conditions generally related to neuropathic symptoms. Treatment for peripheral neuropathy is not currently covered under the James Zadroga 9/11 Health and Compensation Act. Therefore, if confirmed by future studies, our research may have policy implications for consideration of neuropathy as an addition to the list of covered conditions.

## Figures and Tables

**Table 1:** Demographic characteristics among individuals 40 years and older (7 June, 2017–6 April, 2019).

Variable	Indicated ^†^ (*N* = 2059)	Non-Indicated (*N* = 7180)
Age on 9/11, y ^a^	42.1 ± 7.6	39.0 ± 7.3
Age at exam, y ^a^	59.0 ± 7.6	56.0 ± 7.3
Sex, *N* (%)		
Male	1997 (97.0)	7022 (97.8)
Female	62 (3.0)	158 (2.2)
Race, *N* (%)		
Non-Hispanic white	1778 (86.4)	6512 (90.7)
Non-Hispanic black	120 (5.8)	264 (3.7)
Hispanic	141 (6.9)	372 (5.2)
Other	20 (1.0)	32 (0.5)
Work assignment on 9/11, *N* (%)		
Firefighter	1666 (80.9)	6484 (90.3)
EMS	393 (19.1)	696 (9.7)
Smoking status, *N* (%) ^b^		
Never	1453 (70.6)	5625 (78.3)
Former	519 (25.2)	1342 (18.7)
Current	87 (4.2)	213 (3.0)
WTC exposure level, *N* (%)		
High	383 (18.6)	1155 (16.1)
Moderate	1356 (65.9)	5047 (70.3)
Low	320 (15.5)	978 (13.6)
Chronic Alcohol Abuse ^c^		
Yes	0 (0.0)	107 (1.5)
No	2059 (100.0)	7073 (98.5)
PN Risk Factor		
Diabetes (glucose ≥ 140 mg/dL)	1147 (55.7)	-
Cancer (not ‘in situ’)	886 (43.0)	-
Autoimmune Disease	184 (8.9)	-

PN = peripheral neuropathic symptoms; 9/11 = 11 September, 2001; WTC = World Trade Center. ^†^ Rescue/recovery workers with any of the following conditions: diabetes, cancer (not including in situ cancers or non-melanoma skin cancers), or an autoimmune disease. ^a^ Mean ± SD. ^b^ Value at the time of the physical health questionnaire. ^c^ Scored positive on the Alcohol Use Disorders Identification Test (AUDIT) on all questionnaires after the first indication.

**Table 2:** The Diabetic Neuropathy Symptom (DNS) score prevalence among the Indicated and Non-Indicated peripheral neuropathy groups.

DNS Item	Indicated ^†^, *N* = 2059 (%)	Non-Indicated, *N* = 7180 (%)
1. Considering any time in the last two weeks (excluding during exercise), are you suffering of unsteadiness in walking?	184 (8.9)	419 (5.8)
2. Considering any time in the last two weeks (excluding during exercise), do you have a burning, aching pain or tenderness at your legs or feet?	416 (20.2)	1091 (15.2)
3. Considering any time in the last two weeks (excluding during exercise), do you have prickling sensations at your legs and feet?	344 (16.7)	817 (11.4)
4. Considering any time in the last two weeks (excluding during exercise), do you have places of numbness on your legs or feet?	372 (18.1)	954 (13.3)
5. Any of the Above	630 (30.6)	1711 (23.8)

^†^ Rescue/recovery workers with any of the following conditions: diabetes, cancer (not including in situ cancers or non-melanoma skin cancers), or an autoimmune disease.

**Table 3:** Reports of DNS positive scores and paresthesias of the upper and lower extremities in World Trade Center-exposed workers from 7 June, 2017 to 4 April, 2019.

Variable	DNS Positive OR (95% CI)	Paresthesias ^†^		
		Legs/Feet OR (95% CI)	Arms/Hands OR (95% CI)	Legs/Feet and Arms/Hands OR (95% CI)
WTC exposure level				
High	1.35 (1.10, 1.65)	1.36 (0.99, 1.87)	1.47 (1.04, 2.08)	2.47 (1.35, 4.53)
Moderate	1.17 (0.99, 1.38)	1.30 (1.00, 1.70)	1.33 (0.99, 1.78)	2.18 (1.27, 3.75)
Low	Ref.	Ref.	Ref.	Ref.
Age at exam	1.04 (1.03, 1.04)	1.03 (1.02, 1.04)	1.02 (1.00, 1.03)	1.03 (1.01, 1.05)
Race				
Non-Hispanic white	0.97 (0.78, 1.19)	1.03 (0.74, 1.42)	0.93 (0.66, 1.32)	0.77 (0.47, 1.26)
Non-white	Ref.	Ref.	Ref.	Ref.
Sex				
Male	0.64 (0.44, 0.95)	0.41 (0.24, 0.70)	0.46 (0.26, 0.84)	0.36 (0.17, 0.77)
Female	Ref.	Ref.	Ref.	Ref.
Work assignment on 9/11				
EMS	1.05 (0.84, 1.33)	0.91 (0.63, 1.33)	0.81 (0.54, 1.22)	1.14 (0.63, 2.03)
Firefighter	Ref.	Ref.	Ref.	Ref.
Smoking status ^a^				
Ever	1.22 (1.07, 1.39)	1.20 (0.99, 1.46)	1.02 (0.82, 1.28)	1.16 (0.83, 1.61)
Never	Ref.	Ref.	Ref.	Ref.
Chronic Alcohol Abuse ^b^				
Yes	2.32 (1.57, 3.42)	1.48 (0.84, 2.63)	1.43 (0.76, 2.69)	1.99 (0.86, 4.62)
No	Ref.	Ref.	Ref.	Ref.

OR = odds ratio; CI = confidence interval; EMS = emergency medical service worker; Ref. = reference group; DNS = Diabetic Neuropathy Symptom score; 9/11 = 11 September, 2001. ^†^ Often/almost continuously experiencing prickling, pins and needles, burning, aching pain or tenderness in the area defined. ^a^ At the time the questionnaire was taken. ^b^ Scored positive on the Alcohol Use Disorders Identification Test (AUDIT) on all questionnaires after the first indication.

**Table 4:** Multivariable logistic regression using the NHANES 2003–2004 non-diabetic cohort as a national referent population.

Title	OR (95% CI) ^†^
Model 1: WTC exposure overall	
Exposed	2.06 (1.65, 2.57)
Unexposed NHANES	Ref.
Model 2: WTC exposure level	
High	2.36 (1.84, 3.04)
Moderate	2.04 (1.62, 2.56)
Low	1.82 (1.41, 2.35)
Unexposed NHANES	Ref.

NHANES = National Health and Nutrition Examination Survey; WTC = World Trade Center; 9/11 = 11 September, 2001; Ref. = reference. ^†^ Exposure measurements adjusted for age at exam, sex, and race (non-Hispanic white, other).
